# Urinary epidermal growth factor predicts complete remission of proteinuria in Chinese children with IgA nephropathy

**DOI:** 10.1038/s41390-023-02542-0

**Published:** 2023-03-02

**Authors:** Jianmei Zhou, Shuzhen Sun, Dongfeng Zhang, Jianhua Mao, Huijie Xiao, Yong Yao, Fang Wang, Lichun Yu, Ling Liu, Chunyue Feng, Chenglong Li, Baige Su, Hongwen Zhang, Xiaoyu Liu, Ke Xu, Wenjun Ju, Xuhui Zhong, Jie Ding

**Affiliations:** 1 Department of Pediatrics, Peking University First Hospital, Beijing, China; 2 Department of Pediatric Nephrology and Rheumatism and Immunology, Shandong Provincial Hospital, Cheeloo College of Medicine, Shandong University, Jinan, China; 3 Division of Nephrology, Children’s Hospital of Hebei Province, Shijiazhuang, China; 4 Division of Nephrology, The Children’s Hospital, Zhejiang University School of Medicine, National Clinical Research Center for Child Health, Hangzhou, China; 5 Department of Pediatric Nephrology and Rheumatism and Immunology, Shandong Provincial Hospital Affiliated to Shandong First Medical University, Jinan, China; 6 Peking University Clinical Research Institute, Peking University First Hospital, Beijing, China; 7 Department of Internal Medicine, University of Michigan, Ann Arbor, MI USA

## Abstract

**Background:**

This study investigated the association between urinary epidermal growth factor (EGF) and complete remission (CR) of proteinuria in children with IgA nephropathy (IgAN).

**Methods:**

We included 108 patients from the Registry of IgA Nephropathy in Chinese Children. The urinary EGF at the baseline and follow-up were measured and normalized by urine creatinine (expressed as uEGF/Cr). The person-specific uEGF/Cr slopes were estimated using linear mixed-effects models for the subset of patients with longitudinal data of uEGF/Cr. Cox models were used to analyze the associations of baseline uEGF/Cr and uEGF/Cr slope with CR of proteinuria.

**Results:**

Patients with high baseline uEGF/Cr were more likely to achieve CR of proteinuria (adjusted HR 2.24, 95% CI: 1.05–4.79). The addition of high baseline uEGF/Cr on the traditional parameters significantly improved the model fit for predicting CR of proteinuria. In the subset of patients with longitudinal data of uEGF/Cr, high uEGF/Cr slope was associated with a higher likelihood of CR of proteinuria (adjusted HR 4.03, 95% CI: 1.02–15.88).

**Conclusions:**

Urinary EGF may be a useful noninvasive biomarker for predicting and monitoring CR of proteinuria in children with IgAN.

**Impact:**

- High levels of baseline uEGF/Cr (>21.45 ng/mg) could serve as an independent predictor for CR of proteinuria.
- The addition of baseline uEGF/Cr on the traditional clinical pathological parameters significantly improved the fitting ability for the prediction of CR of proteinuria.
- Longitudinal data of uEGF/Cr were also independently associated with CR of proteinuria.
- Our study provides evidence that urinary EGF may be a useful noninvasive biomarker in the prediction of CR of proteinuria as well as monitoring therapeutic response, thus guiding treatment strategies in clinical practice for children with IgAN.

## Introduction

IgA nephropathy (IgAN) is one of the most common primary glomerular diseases in children, with various clinical manifestations ranging from asymptomatic microscopic hematuria to rapidly progressive kidney failure.^1^ Approximately 30–40% of patients would progress to end-stage kidney disease within 20 years from the onset of the disease.^2^ Proteinuria has been identified as one of the predictors of poor kidney outcomes.^3^ Reduction of proteinuria is one of the most critical indicators for therapeutic response, especially in children with IgAN, who even initially present an increase of estimated glomerular filtration (eGFR) during childhood.^4^ Proteinuria reduction as one of the endpoints in clinical trials for IgAN increasingly received more attention in recent years.^5^ Previous data revealed that complete remission (CR) of proteinuria was significantly associated with better long-term prognosis for patients with IgAN,^6–8^ indicating that achievement of CR of proteinuria should be considered as one of the critical goals in treatment strategies for IgAN. However, patients with IgAN show large heterogeneity in proteinuria remission. Some patients achieve spontaneous proteinuria remission without receiving medications,^9^ while some patients present persistent massive proteinuria even after therapy of glucocorticoids and/or non-glucocorticoid immunosuppressants.^10^ So far, several traditional clinical and histological parameters, including the quantification of proteinuria, hypertension, eGFR, and Oxford classification, are unable to identify patients with less likelihood of achieving CR of proteinuria. Novel noninvasive biomarkers were scarce. Thus, there is a demand to identify novel biomarkers to help with the prediction of CR of proteinuria, especially since it was not known whether CR of proteinuria could be achieved until several months after treatment.

Urinary epidermal growth factor (EGF) is mainly derived from its synthesis and secretion of the thick ascending limb of the loop of Henle and the distal convoluted tubule.^11^ It reflects the functional tubular mass and repairs tubular epithelial cells by acting on EGF receptors.^12^ Multiple studies have reported its significant decrease in adult and pediatric kidney disease.^13,14^ More importantly, its predictive value for kidney outcomes has been reported in adults with a variety of kidney diseases (including IgAN)^14–20^ and children with Alport syndrome,^13^ CKD,^21^ primary nephrotic syndrome^22^ and type 1 diabetes.^23^ Furthermore, high EGF was reported as a predictor for CR of proteinuria in adults with primary glomerulonephritis^24^ and lupus nephritis.^25^ However, pediatric patients differed significantly from adults.^26^ Children with IgAN showed significantly better kidney survival than adult patients.^26,27^ As such, it is necessary to explore the predictive value of urinary EGF for CR of proteinuria in children with IgAN. Here, we aimed to investigate urinary EGF as a predictor for CR of proteinuria.

## Materials and methods

### Patients and study design

The patients were selected from the Registry of IgA Nephropathy in Chinese Children (RACC, registration no. in clinicaltrials.gov: NCT03015974). RACC cohort prospectively enrolled children with IgAN from 28 centers across China. Clinical data, pathological classification, medications prescribed, and urine samples were prospectively collected from participants after enrollment into the RACC study and during follow-up. In this study, patients were selected according to the following inclusion criteria: (1) age <18 years; (2) biopsy-proven IgAN; (3) urine supernatant samples available; and (4) 24-h urinary protein (24-h UP) >150 mg/d and urine protein: creatinine ratio >0.2 g/g at baseline. Patients were excluded when their IgA deposition in glomeruli was suspected to be secondary to the other diseases. The baseline in this study was defined as the date of the first urine sample collection for each patient.

### Data extraction and clinical assessment

Clinical data and pathological data were extracted from the RACC database, including age, sex, disease history, physical examination (blood pressure, height, and weight), laboratory parameters, Oxford classification (MEST-C scores), the intensity of IgA staining, and drug usage. Laboratory parameters included 24-h UP, urine protein creatinine ratio (uPCR), urine blood cell count (uRBC), serum creatinine, serum IgG levels, serum IgA levels, serum IgM levels, serum C3 levels, and serum C4 levels. The eGFR was calculated using the bedside serum creatinine-based estimating equation for children.^28^ Patients with a history of hypertension or blood pressure above the 95th percentile of age- and sex-matched Chinese healthy children^29^ were categorized as having hypertension. The presence of acute kidney diseases (AKD) was evaluated according to the 2012 KDIGO guidelines.^30^ Drug usage at baseline for patients was classified into the following four categories mainly based on the 2016 Chinese guidelines for children with IgAN:^31^ (1) ACEi/ARBs only; (2) glucocorticoids; (3) non-glucocorticoid immunosuppressants; and (4) glucocorticoids plus non-glucocorticoid immunosuppressants.

### Urine sample collection and measurement of urinary EGF

Approximately 50 ml of spot urine at baseline and during the follow-up was collected from patients and centrifuged at 4000 rpm for 15 min at 4 °C, and the supernatant was aliquoted and stored at –80 °C until the batch test. The measurement of urinary EGF levels was performed according to the protocol, as described previously.^13^ The concentrations of EGF were determined using Human EGF Quantikine ELISA kits (R&D Systems, Minneapolis, MN) according to the manufacturer’s protocol. Standards and quality controls of the high, medium, and low concentrations (R&D Systems, Minneapolis, MN) were used in each batch test. All analyses were conducted in duplicate. The intra-assay and inter-assay coefficients of variation evaluated by quality controls were less than 8% and 12%, respectively. The concentrations of EGF were normalized by urine creatinine concentration (expressed as uEGF/Cr) to adjust for individuals’ hydration status.

### Statistical analyses

Normal distribution for variables was tested by the Kolmogorov–Smirnov test. Continuous variables were expressed as mean with standard deviation for normal distribution and medians with interquartile ranges for non-normal distribution. Categorical variables were described as frequencies and percentages. Comparison for continuous variables was performed using the Student *t*-test, Mann–Whitney *U* test, or Kruskal–Wallis tests as appropriate. *χ*^2^ test or Fisher’s exact test was used for the comparison of categorical variables. Log2 transformation was applied to uEGF/Cr to reduce skewness. Partial Pearson correlation analysis was used to test the correlations between baseline transformed uEGF/Cr (log2) and clinical continuous variables at baseline, controlling for patients’ age.

The endpoint of this study was CR of proteinuria, defined as 24-h UP ≤150 mg/d or uPCR ≤0.2 g/g on two consecutive urinalyses.^6^ Time to event was defined as the time to the first achievement of CR of proteinuria during the follow-up. The association between the baseline uEGF/Cr on a continuous scale and CR of proteinuria was evaluated by restricted cubic splines (RCS), which has been widely used to assess and visualize the non-linear relationship between continuous variables and dependent variables.^32^ The baseline uEGF/Cr concentrations transformed using RCS were incorporated into Cox regression model to explore the relationship with CR of proteinuria. The reference value (HR = 1) was set at the median of uEGF/Cr, and the knots were located at the 5th, 35th, 65th, and 95th percentiles.^33^ Maximally selected rank statistics were used to identify the optimal cutoff value of baseline uEGF/Cr.^34,35^ For every potential cutoff point, the standardized log-rank statistic was computed. The cutoff point that provided the best separation of the endpoint of CR of proteinuria into two groups and generated maximal standardized statistics, was identified as the optimal cutoff point. Then, patients were classified into the high baseline uEGF/Cr group and the low baseline uEGF/Cr group based on the optimal cutoff value. Kaplan–Meier analysis was used to estimate the incidence of CR of proteinuria. To adjust for the potential effect of the covariates, univariate Cox regression models were initially performed to assess the association between the CR of proteinuria and each candidate variable, including age, sex, 24-h UP, eGFR, serum albumin, time from onset, presence of AKD, gross hematuria, hypertension, serum IgG, serum IgA, serum IgM, serum C3, serum C4, Oxford classification (M score, E score, S score, and C score), and drug usage. Variables with a *p* value <0.10 in the univariate analysis entered the multivariate Cox analysis to fit the prediction model of CR of proteinuria. Nested Cox regression models were used to explore the role of the addition of uEGF/Cr on the traditional clinical and pathological parameters for predicting CR of proteinuria.^17^ The fitting capacity of models was assessed by *R* square, C-statistics, and Akaike information criterion (AIC). The improved predictive ability of additional uEGF/Cr was tested using the likelihood ratio tests.

The subset of patients who had last uEGF/Cr measurement over 12 months from baseline were selected to further investigate the associations of longitudinal change of uEGF/Cr and CR of proteinuria. Linear mixed-effects models with random intercepts and random slopes were used to estimate the person-specific uEGF/Cr slope. Patients were further divided into the high uEGF/Cr slope group and the low uEGF/Cr slope group according to the median of uEGF/Cr slope. Cox regression models were used to investigate the associations between uEGF/Cr slope and CR of proteinuria.

In addition, sensitivity analysis was performed to examine the robustness of the results. Firstly, we reanalyzed the association of baseline uEGF/Cr for CR of proteinuria after imputing the missing data by multiple imputation with ten imputation datasets, based on variables as follows: age, sex, uEGF/Cr, 24-h UP, eGFR, serum albumin, time from onset, presence of AKD, hypertension, gross hematuria, Oxford classification (MEST-C scores), drug usage, serum IgG, IgA, IgM, C3, and C4 levels. Pooled data of ten imputation datasets were analyzed. Secondly, we observed whether the results would change if patients with the presence of AKD were excluded. Thirdly, we reanalyzed whether the results would change if patients who had prior therapy were excluded.

All analyses were conducted using R (4.0.1). The results were considered statistically significant when two-tailed *p* values were less than 0.05.

## Results

### Patient characteristics

A total of 108 patients with 160 urine samples were eligible for this study. Table 1 shows the clinical and pathological characteristics for patients. At baseline, patients had an average age of 10.4 ± 2.8 years, with male to female ratio of 1.8. Most of the patients (*n* = 99, 91.7%) were prescribed glucocorticoids or non-glucocorticoid immunosuppressants. Most baseline urine samples (*n* = 105, 97.2%) were collected on the early morning of the kidney biopsy or within 1 month after the kidney biopsy.

**Table 1:** Clinical and pathological characteristics at baseline.

	All (*n* = 108)	Low uEGF/Cr (*n* = 49)	High uEGF/Cr (*n* = 59)	*p* value^a^
Age (years)	10.4 ± 2.8	11.4 ± 2.3	9.6 ± 3.0	0.001
Sex (male)	69 (63.9%)	38 (77.6%)	31 (52.5%)	0.013
uEGF/Cr (ng/mg)	24.0 (15.8–34.9)	15.2 (10.2–18.9)	34.5 (28.2–44.1)	<0.001
24-h UP (mg/kg)	55.9 ± 44.5	53.3 ± 45.0	58.2 ± 44.3	0.572
uPCR (g/g)	1.9 (0.8–4.1)	1.9 (0.6–3.2)	1.9 (0.9–4.5)	0.618
eGFR (ml/min/1.73 m^2^)	98.1 ± 37.4	77.3 ± 27.1	115 ± 36.0	<0.001
Serum albumin (g/l)	33.4 ± 8.4	34.0 ± 7.2	33.0 ± 9.3	0.561
uRBC (per ul)	540 (161–1886)	851 (174–1896)	456 (149–1830)	0.243
Time from onset (months)	1.3 (0.8–3.0)	1.0 (0.7–2.0)	1.6 (0.9–4.6)	0.127
Gross hematuria	90 (83.3%)	43 (87.8%)	47 (79.7%)	0.387
Hypertension	47 (43.5%)	20 (40.8%)	27 (45.8%)	0.748
Presence of AKD	22 (20.4%)	19 (38.8%)	3 (5.08%)	<0.001
Prior therapy	8 (7.4%)	4 (8.2%)	4 (6.8%)	1.000
Serum IgG (g/l)^b^	6.64 ± 3.44	7.76 ± 3.65	5.69 ± 2.97	0.003
Serum IgA (g/l)^b^	2.30 ± 1.12	2.64 ± 1.10	2.02 ± 1.07	0.004
Serum IgM (g/l)^b^	1.23 ± 0.53	1.14 ± 0.41	1.31 ± 0.61	0.103
Serum C3 (g/l)^b^	1.08 ± 0.26	1.04 ± 0.26	1.11 ± 0.26	0.144
Serum C4 (g/l)^b^	0.25 ± 0.09	0.26 ± 0.09	0.24 ± 0.10	0.308
IgA staining				0.600
2+	14 (13.0%)	4 (8.16%)	10 (16.9%)	
2+~3+	9 (8.33%)	4 (8.16%)	5 (8.47%)	
3+	52 (48.1%)	26 (53.1%)	26 (44.1%)	
3+~4+	17 (15.7%)	9 (18.4%)	8 (13.6%)	
4+	16 (14.8%)	6 (12.2%)	10 (16.9%)	
Oxford classification				
M1^b^	82 (84.5%)	37 (80.4%)	45 (88.2%)	0.435
E1^b^	66 (68.0%)	13 (28.3%)	18 (35.3%)	0.600
S1^b^	32 (33.0%)	16 (34.8%)	16 (31.4%)	0.888
T1/2^b^	2 (2.06%)	2 (4.08%)	0 (0.00%)	0.222
C1/2	68 (63.0%)	32 (65.3%)	36 (61.0%)	0.627
Drug usage^b^				0.971
ACEi/ARBs only	7 (6.60%)	3 (6.25%)	4 (6.90%)	
Glucocorticoids	27 (25.5%)	13 (27.1%)	14 (24.1%)	
Non-glucocorticoid immunosuppressants^c^	8 (7.55%)	4 (8.33%)	4 (6.90%)	
Non-glucocorticoid immunosuppressants^c^ plus glucocorticoids	64 (60.4%)	28 (58.3%)	36 (62.1%)	
Follow-up (months)	18.7 ± 14.0	19.6 ± 15.5	18.0 ± 12.8	0.575

Continuous variables are reported as mean ± SD for normal distribution and median (interquartile range) for non-normal distribution. Categorical variables are reported as *n* (%).

*24-h UP* 24-h urinary protein, *uPCR* urine protein creatinine ratio, *eGFR* estimated glomerular filtration rate, *uRBC* urine red blood cell count, *AKD* acute kidney disease, *ACEi/ARBs* angiotensin-converting enzyme inhibitors/angiotensin receptor blockers.

^a^Comparison for two groups.

^b^With missing values for serum IgG (*n* = 6), serum IgA (*n* = 1), serum IgM (*n* = 6), serum C3 (*n* = 2), serum C4 (*n* = 2), M score (*n* = 11), E score (*n* = 11), S score (*n* = 11), T score (*n* = 11), and drug usage (*n* = 2).

^c^Non-glucocorticoid immunosuppressants in this study included mycophenolate mofetil, cyclophosphamide, cyclosporine A, tacrolimus, and leflunomide.

The baseline uEGF/Cr of 21.45 ng/mg was identified as the optimal cutoff value by using maximally selected log-rank statistics (Supplementary Fig. S1). Patients with baseline uEGF/Cr >21.45 ng/mg were classified as the high baseline uEGF/Cr group and those with baseline uEGF/Cr ≤21.45 ng/mg as the low baseline uEGF/Cr group. Further comparison of patient characteristics between the two groups showed that patients in the high baseline uEGF/Cr group were at a younger age, had higher eGFR, lower levels of serum IgG and serum IgA, and a lower percentage of AKD. During follow-up, 24 patients had the last uEGF/Cr measurement over 12 months from baseline. A total of 76 urine samples were collected from this subset of patients.

### Correlations of baseline uEGF/Cr with clinical and pathological parameters at baseline

**Fig. 1:**
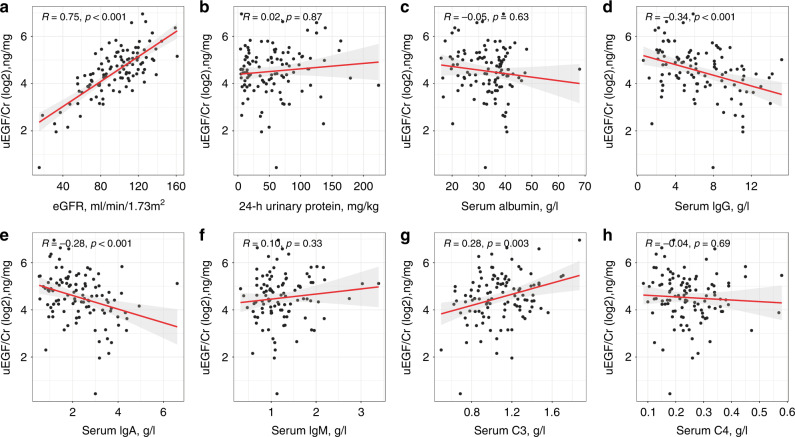
The correlations between uEGF/Cr (log2) and clinical as well as serum immunological parameters. The correlation of uEGF/Cr with eGFR (**a**), 24-h urinary protein (**b**), serum albumin (**c**), serum IgG (**d**), serum IgA (**e**), serum IgM (**f**), serum C3 (**g**), and serum C4 (**h**), respectively. All *r* values were adjusted by patient age.

**Fig. 2:**
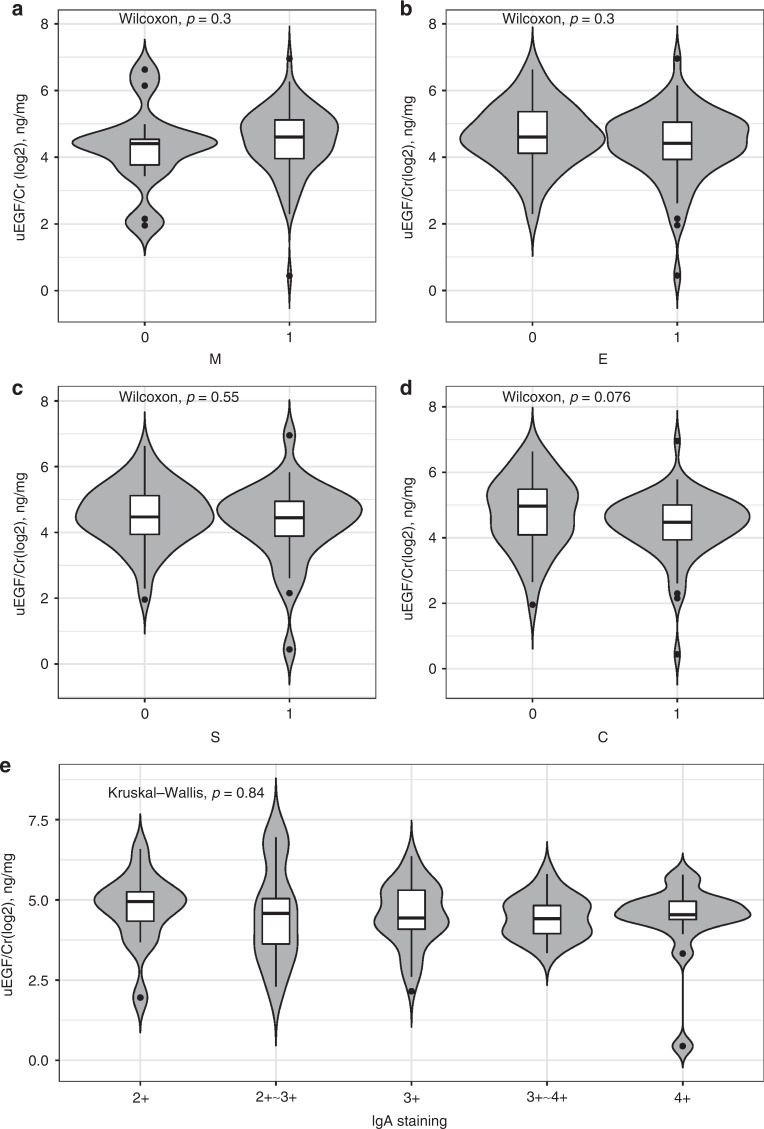
The correlations between uEGF/Cr (log2) and pathological parameters. The violin plots for the comparisons of uEGF/Cr in terms of Oxford MEST-C classification (between M0 and M1 (**a**), E0 and E1 (**b**), S0 and S1 (**c**), C0 and C1/2 (**d**)) and the intensity of IgA staining in kidney tissue (**e**).

The levels of uEGF/Cr at baseline were significantly positively correlated with eGFR after adjusting for age (Fig. 1a). No significant correlation was found between uEGF/Cr and 24-h UP (Fig. 1b) or serum albumin (Fig. 1c). Correlations between uEGF/Cr and serum immunological parameters showed that uEGF/Cr was significantly negatively correlated with serum IgG (Fig. 1d) and IgA levels (Fig. 1e), but not correlated with serum IgM levels (Fig. 1f). Moreover, uEGF/Cr showed a significantly positive correlation with serum C3 levels (Fig. 1g), but no correlation with serum C4 levels (Fig. 1h). Comparison for the levels of uEGF/Cr in terms of Oxford classification (MEST-C scores) showed that there were no correlations between uEGF/Cr levels and the presence of mesangial hypercellularity, endocapillary proliferation, segmental glomerulosclerosis, or crescents formation, respectively (Fig. 2a–d). Also, no correlation was found between uEGF/Cr and the intensity of IgA staining in kidney tissue (Fig. 2e).

### The association of baseline uEGF/Cr with CR of proteinuria

**Fig. 3:**
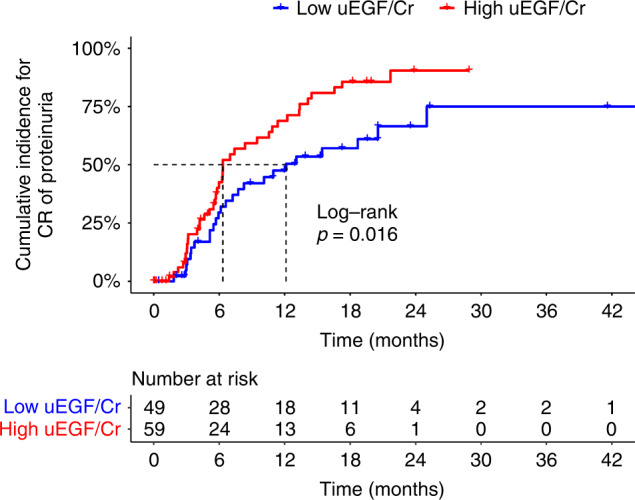
Kaplan–Meier curves for CR of proteinuria. High uEGF/Cr:uEGF/Cr > 21.45 ng/mg. Low uEGF/Cr:uEGF/Cr ≤ 21.45 ng/mg. CR complete remission.

During the average follow-up of 18.7 months, 64 (59.3%) patients achieved CR of proteinuria. In Supplementary Fig. S2, the RCS showed there was a non-linear association between baseline uEGF/Cr and CR of proteinuria (S-shaped, *p* for non-linearity = 0.024). After patients were divided into two groups based on the optimal cutoff value of 21.45 ng/mg, the median time to CR of proteinuria for the two groups showed a significant difference, with 6.3 months for the high baseline uEGF/Cr group and 12.1 months for the low baseline uEGF/Cr group, respectively (Fig. 3, log-rank test *p* = 0.016).

As shown in Table 2, univariate Cox regression analysis revealed that patients in the high baseline uEGF/Cr group were more likely to achieve CR of proteinuria (unadjusted HR = 1.85, 95% CI: 1.11–3.06, *p* = 0.018). After adjusting for covariates screened by univariate analysis, patients with high baseline uEGF/Cr could still achieve an adjusted 2.24-fold higher likelihood for CR of proteinuria (95% CI: 1.05–4.79, *p* = 0.037), compared with those with low baseline uEGF/Cr. In addition, age, 24-h UP, and S score were also found to be independently associated with CR of proteinuria in the multivariate Cox regression model. Furthermore, the results of nested Cox models (Table 3) showed that the addition of uEGF/Cr on the models consisting of traditional clinical and pathological parameters resulted in increased C-statistics and R squares, and decreased AIC values, suggesting the improvements in model fit for predicting CR of proteinuria.

**Table 2:** Univariate and multivariate Cox regression models for CR of proteinuria.

Characteristics	Univariate		Multivariate	
	HR (95% CI)	*p* value	HR (95% CI)	*p* value
Age	0.85 (0.78–0.93)	<0.001	0.81 (0.72–0.92)	0.001
Sex (male vs female)	1.02 (0.61–1.69)	0.942	–	–
eGFR (log2)	1.08 (0.69–1.70)	0.742	–	–
24-h UP (log2)	0.79 (0.65–0.94)	0.010	0.61 (0.44–0.85)	0.004
Serum albumin	1.04 (1.01–1.08)	0.012	0.94 (0.88–1.01)	0.113
Time from onset	0.99 (0.96–1.02)	0.630	–	–
uRBC (log2)	1.05 (0.96–1.16)	0.291	–	–
Goss hematuria (yes vs no)	2.91 (1.25–6.76)	0.013	2.43 (0.86–6.9)	0.094
Hypertension (yes vs no)	0.68 (0.41–1.12)	0.132	–	–
Presence of AKD	1.18 (0.68–2.06)	0.557	–	–
Prior therapy	0.49 (0.18–1.36)	0.174	–	–
IgG (log2)	1.4 (1.03–1.89)	0.031	2.09 (1.24–3.52)	0.005
IgA (log2)	1.24 (0.91–1.69)	0.179	–	–
IgM (log2)	1.25 (0.83–1.88)	0.281	–	–
C3 (log2)	1.3 (0.65–2.58)	0.462	–	–
C4 (log2)	1.28 (0.82–1.99)	0.278	–	–
Drug usage				
ACEi/ARBs only	0.55 (0.08–4.04)	0.558	0.33 (0.04–2.64)	0.293
Glucocorticoids	1.89 (1.09–3.27)	0.023	1.33 (0.65–2.74)	0.432
Non-glucocorticoid immunosuppressants	0.98 (0.35–2.77)	0.977	0.48 (0.15–1.57)	0.226
Non-glucocorticoid immunosuppressants plus glucocorticoids	Reference	–	Reference	–
Oxford classification				
M1	0.92 (0.47–1.83)	0.818	–	–
E1	1.63 (0.89–2.99)	0.113	–	–
S1	0.53 (0.3–0.95)	0.032	0.5 (0.26–0.96)	0.036
C1/2	1.55 (0.9–2.68)	0.114	–	–
uEGF/Cr (high vs low)	1.85 (1.11–3.06)	0.018	2.24 (1.05–4.79)	0.037

**Table 3:** Nested Cox models for the associations of baseline uEGF/Cr with CR of proteinuria.

Models	HR (95% CI) (high vs low)	C-index	AIC	*R*^2^	*p* value for likelihood ratio test
Crude model	1.85 (1.11–3.06)	–	–	–	–
Model 1	–	0.695	473.92	0.218	–
Model 1 + uEGF/Cr	2.07 (1.01–4.24)	0.710	471.90	0.247	0.045 (vs Model 1)
Model 2	–	0.725	474.42	0.271	–
Model 2 + uEGF/Cr	3.05 (1.41–6.61)	0.743	468.09	0.325	0.004 (vs Model 2)
Model 3	–	0.740	409.33	0.370	–
Model 3 + uEGF/Cr	2.78 (1.19–6.49)	0.763	405.55	0.406	0.016 (vs Model 3)
Model 4	–	0.749	414.40	0.382	–
Model 4 + uEGF/Cr	2.74 (1.18–6.35)	0.765	410.66	0.418	0.017 (vs Model 4)

Crude model: did not adjust for any covariate.

Model 1 adjusted for age, sex, 24-h urinary protein, eGFR and serum albumin.

Model 2 further adjusted for gross hematuria, hypertension, time from onset, and presence of AKD.

Model 3 further adjusted for Oxford classification (M, E, S, and C).

Model 4 further adjusted for drug usage.

### The association of uEGF/Cr slope with CR of proteinuria

In the subset of 24 patients who had last uEGF/Cr measurement over 12 months from the baseline, the median uEGF/Cr slope was 2.57 (0.54–3.73) ng/mg/year. Patients with uEGF/Cr slope >2.57 ng/mg/year were classified as the high uEGF/Cr slope group and those with uEGF/Cr slope ≤2.57 ng/mg/year as the low uEGF/Cr slope group. No significant difference was found in the baseline uEGF/Cr, age, sex, baseline 24-h UP, and eGFR between the two groups (Table 4). The high uEGF/Cr slope was significantly associated with a greater likelihood of achieving CR of proteinuria (Table 5, adjusted HR 4.03, 95% CI: 1.02–15.88, *p* = 0.046).

**Table 4:** Clinical characteristics for the subset of patients with longitudinal data of uEGF/Cr measurements.

	All (*n* = 24)	Low uEGF/Cr slope (*n* = 12)	High uEGF/Cr slope (*n* = 12)	*p* value^a^
Age at baseline (years)	11.1 ± 2.98	11.8 ± 2.57	10.5 ± 3.33	0.386
Sex (male)	18 (75.0%)	10 (83.3%)	8 (66.7%)	0.640
24-h UP at baseline (mg/kg)	45.4 ± 31.1	56.0 ± 35.3	34.7 ± 22.8	0.133
eGFR at baseline (ml/min/1.73 m^2^)	95.7 ± 33.1	99.9 ± 32.9	91.5 ± 34.3	0.488
uEGF/Cr at baseline (ng/mg)	20.7 (12.3–29.7)	21.0 (14.5–31.2)	18.3 (11.5–23.9)	0.488
uEGF/Cr slope (ng/mg/year)	2.57 (0.54–3.73)	0.51 (–1.13 to 0.89)	3.93 (3.35–6.29)	<0.001
Follow-up (months)	26.9 ± 14.1	30.6 ± 16.4	23.2 ± 10.7	0.356

Continuous variables are reported as mean ± SD for normal distribution and median (interquartile range) for non-normal distribution.

^a^Comparison between the low uEGF/Cr slope group and the high uEGF/Cr slope group.

**Table 5:** The associations between uEGF/Cr slope and CR of proteinuria.

	Patients at risk	Univariate		Multivariate^a^	
		HR (95% CI)	*p* value	HR (95% CI)	*p* value
Low uEGF/Cr slope	12	Reference	–	Reference	–
High uEGF/Cr slope	12	3.96 (1.29–12.17)	0.016	4.03 (1.02–15.88)	0.046

^a^Adjusted for baseline uEGF/Cr (high, low), age, sex, and 24-h UP.

### Sensitivity analysis

First, the results remained robust after missing values were imputed using multiple imputation (Supplementary Table S1, adjusted HR 2.92, 95% CI: 1.44–5.94). Second, after patients with AKD were excluded, the high baseline uEGF/Cr group still showed a higher likelihood for the achievement of CR of proteinuria than the low baseline uEGF/Cr group (Supplementary Table S2, adjusted HR 3.68, 95% CI: 1.31–10.28). After patients with prior therapy were excluded, patients with high baseline uEGF/Cr were still more likely to achieve CR of proteinuria (Supplementary Table S3, adjusted HR 1.96, 95% CI: 0.89–4.28), although the result was borderline significant (*p* = 0.093).

## Discussion

Our results investigated the association of uEGF/Cr with CR of proteinuria in children with IgAN. We observed that there was a non-linear association between baseline uEGF/Cr and CR of proteinuria. Patients with high baseline uEGF/Cr (>21.45 ng/mg) showed a higher likelihood of achieving CR of proteinuria than those with low baseline uEGF/Cr levels (≤21.45 ng/mg), indicating the role of baseline uEGF/Cr as an independent predictor for CR of proteinuria during the follow-up. This was similar to the results reported in adults with primary glomerulonephritis^24^ and lupus nephritis.^25^ This result indicates that uEGF/Cr could be used to prospectively identify patients with potentially unfavorable therapeutic responses, thus guiding treatment strategies for IgAN in children.

We also examined the additive effect of uEGF/Cr for the model fit. It was observed that there were improvements of model fit when uEGF/Cr was added to the models consisting of traditional parameters. This result supports uEGF/Cr as a valuable biomarker to help with a more accurate prediction of CR of proteinuria in children with IgAN.

More importantly, we calculated the slope for uEGF/Cr to further investigate the association of longitudinal change of uEGF/Cr with CR of proteinuria. We found that a high uEGF/Cr slope (>2.57 ng/mg/year) during the follow-up was significantly associated with a higher likelihood of CR of proteinuria, independent of baseline uEGF/Cr and other commonly used clinical parameters. The role of uEGF/Cr levels over time in proteinuria remission was rarely described previously. Our novel findings suggest there is a potential role of repeated uEGF/Cr measurements in monitoring therapeutic response.

To validate the robustness of our results, sensitivity analysis with three methods was conducted. After missing data were imputed, those with high baseline uEGF/Cr still showed a significantly higher likelihood for CR of proteinuria. After either excluding patients with AKD or those with prior therapy, high baseline uEGF/Cr could still be identified as a predictor for CR of proteinuria. These analyses further elucidated the robustness of the results that the baseline uEGF/Cr serves as an independent predictor for CR of proteinuria during the follow-up.

Some other urinary cytokines, generated by both resident and nonresident kidney cells, also play significant roles in the progression of kidney function for IgAN. These include interleukin 6,^36^ transforming growth factor-β1,^20^ and monocyte chemoattractant protein-1.^19^ However, their urinary concentration is influenced by both glomerular filtration and intrarenal production, which potentially affects their utility.^15^ Unlike the above cytokines, EGF is minimally detectable in plasma, and urinary EGF had highly restricted intrarenal expression.^37^ For this reason, urinary EGF is superior to the other independent predictors in children with IgAN. The underlying mechanism of the predictive value of high urinary EGF levels for CR of proteinuria in children with IgAN might be associated with its biological function. It signals in an autocrine/paracrine way through the EGF receptor, which is involved in cell proliferation, metabolism, differentiation, and survival.^38^ The EGF was found to protect against high glucose-induced podocyte injury by promoting podocyte proliferation and inhibiting podocyte apoptosis.^39^ Exogenous EGF accelerates the regeneration of kidney tubules and the recovery of kidney function in animal models of acute kidney injury.^40^ As such, EGF might play a protective role in the reduction of proteinuria by repairing podocytes and tubular cells. Further study is still needed to investigate the detailed mechanisms of the protective role of urinary EGF on CR of proteinuria.

In addition, the correlations between baseline uEGF/Cr levels and clinical parameters as well as pathological parameters at baseline were investigated in this study. Given that multiple laboratory parameters were reported to present age-related changes,^41–43^ we adjusted for patients’ age in all correlation analyses. The results showed there was a significantly positive correlation between baseline uEGF/Cr levels and baseline eGFR, consistent with previous results.^15,19,36^ The predictive value of baseline uEGF/Cr levels for kidney outcomes has been previously described in adults with IgAN.^14,15,19^ Considering the apparent discrepancy in kidney outcomes between pediatric patients and adult patients with IgAN,^4^ it is notable whether there were associations between uEGF/Cr and the progression of kidney function in children with IgAN. Further investigation is still needed. There was no significant correlation between uEGF/Cr levels and urinary protein in our study, as described by previous studies.^15,19,24^ Similar to the results in adulthood IgAN,^20^ no correlation was observed between uEGF/Cr levels and the presence of mesangial hypercellularity, endocapillary proliferation, segmental glomerulosclerosis, or crescents formation, respectively. Since only two patients were observed with the presence of tubular interstitial fibrosis in our study, the comparison of uEGF/Cr between T0 and T1/2 was not performed. In addition, we observed that uEGF/Cr levels were significantly correlated with the levels of serum IgG, IgA, and C3. These serum immunological parameters were reported to be associated with kidney histological lesions or kidney outcomes of IgAN.^44–50^

Our study had several strengths. On the analytical aspects, the method of ELISA was used in our study, which is considered the gold standard for measuring EGF because of its better sensitivity and specificity compared with other methods (such as radioimmunoassays, radioreceptor assays, enzyme immunoassays, and ultramicroELISA assay) for detection of EGF in urine.^51^ Also, both baseline uEGF/Cr and longitudinal data of uEGF/Cr were measured to investigate their associations with the CR of proteinuria. Furthermore, we revealed high baseline uEGF/Cr could not only serve as an independent predictor, but also generated improvements in model fit for the prediction of CR of proteinuria. Thus, our results provided evidence for uEGF/Cr as a useful noninvasive biomarker to predict proteinuria remission as well as monitor therapeutic response for IgAN in children.

There were some limitations to this study. First, the association of uEGF/Cr with the progression of kidney function was not investigated in this study, due to the relatively slow progression in pediatric patients and short follow-up duration. Longer follow-up was needed to further analyze the association of uEGF/Cr with long-term kidney outcomes in children with IgAN. Second, due to the limited sample size, we did not analyze the association of uEGF/Cr with the therapeutic response of a specific drug. Third, it is still necessary to validate the optimal cutoff value of uEGF/Cr identified by our study for the prediction of CR of proteinuria. The results need to be further validated in another independent cohort with a larger sample size.

## Conclusion

The levels of uEGF/Cr were correlated with commonly used clinical parameters. Our results provide evidence for uEGF/Cr as a promising noninvasive biomarker for predicting CR of proteinuria as well as monitoring therapeutic response.

## Supplementary information

Supplementary Materials

## Data Availability

The datasets generated during and/or analyzed during this study are available from the corresponding author on reasonable request.

## References

[1] Yoshikawa N, Iijima K, Ito H (1999). IgA nephropathy in children. Nephron.

[2] Natale P (2020). Immunosuppressive agents for treating IgA nephropathy. Cochrane Database Syst. Rev..

[3] Kamei K (2016). Proteinuria during follow-up period and long-term renal survival of childhood IgA nephropathy. PLoS One.

[4] Barbour SJ (2021). Updating the international IgA nephropathy prediction tool for use in children. Kidney Int..

[5] Thompson A (2019). Proteinuria reduction as a surrogate end point in trials of IgA nephropathy. Clin. J. Am. Soc. Nephrol..

[6] Tatematsu M (2012). Complete remission within 2 years predicts a good prognosis after methylprednisolone pulse therapy in patients with IgA nephropathy. Clin. Exp. Nephrol..

[7] Reich HN, Troyanov S, Scholey JW, Cattran DC, Toronto Glomerulonephritis Registry. (2007). Remission of proteinuria improves prognosis in IgA nephropathy. J. Am. Soc. Nephrol..

[8] Hotta O (2001). Tonsillectomy and steroid pulse therapy significantly impact on clinical remission in patients with IgA nephropathy. Am. J. Kidney Dis..

[9] Shima Y (2013). Spontaneous remission in children with IgA nephropathy. Pediatr. Nephrol..

[10] Wan QJ (2016). Tacrolimus combined with low-dose corticosteroids is an effective and safe therapeutic option for refractory IgA nephropathy. Exp. Ther. Med..

[11] Gesualdo L (1996). Expression of epidermal growth factor and its receptor in normal and diseased human kidney: an immunohistochemical and in situ hybridization study. Kidney Int..

[12] Fanos V, Pizzini C, Mussap M, Benini D, Pleban M (2001). Urinary epidermal growth factor in different renal conditions in children. Ren. Fail.

[13] Li B (2018). Urinary epidermal growth factor as a prognostic marker for the progression of Alport syndrome in children. Pediatr. Nephrol..

[14] Ranieri E, Gesualdo L, Petrarulo F, Schena FP (1996). Urinary Il-6/EGF ratio: a useful prognostic marker for the progression of renal damage in IgA nephropathy. Kidney Int..

[15] Ju W (2015). Tissue transcriptome-driven identification of epidermal growth factor as a chronic kidney disease biomarker. Sci. Transl. Med..

[16] Wu L (2020). Associations of urinary epidermal growth factor and monocyte chemotactic protein-1 with kidney involvement in patients with diabetic kidney disease. Nephrol. Dial. Transplant..

[17] Wu L (2018). Urinary epidermal growth factor predicts renal prognosis in antineutrophil cytoplasmic antibody-associated vasculitis. Ann. Rheum. Dis..

[18] Stangou M (2009). Urinary levels of epidermal growth factor, interleukin-6 and monocyte chemoattractant protein-1 may act as predictor markers of renal function outcome in immunoglobulin A nephropathy. Nephrology (Carlton).

[19] Torres DD (2008). The ratio of epidermal growth factor to monocyte chemotactic peptide-1 in the urine predicts renal prognosis in IgA nephropathy. Kidney Int..

[20] Segarra-Medrano A (2017). Value of urinary levels of interleukin-6, epidermal growth factor, monocyte chemoattractant protein type1 and transforming growth factor beta1 in predicting the extent of fibrosis lesions in kidney biopsies of patients with IgA nephropathy. Nefrologia.

[21] Azukaitis K (2019). Low levels of urinary epidermal growth factor predict chronic kidney disease progression in children. Kidney Int..

[22] Gipson DS (2020). Urinary epidermal growth factor as a marker of disease progression in children with nephrotic syndrome. Kidney Int. Rep..

[23] Ledeganck KJ (2021). The next generation: urinary epidermal growth factor is associated with an early decline in kidney function in children and adolescents with type 1 diabetes mellitus. Diabetes Res. Clin. Pract..

[24] Chanrat E (2018). Urine epidermal growth factor, monocyte chemoattractant protein-1 or their ratio as predictors of complete remission in primary glomerulonephritis. Cytokine.

[25] Ngamjanyaporn P (2022). Predicting treatment response and clinicopathological findings in lupus nephritis with urine epidermal growth factor, monocyte chemoattractant protein-1 or their ratios. PLoS One.

[26] Cambier A (2020). Clinical and histological differences between adults and children in new onset IgA nephropathy. Pediatr. Nephrol..

[27] Haas M (2008). IgA nephropathy in children and adults: comparison of histologic features and clinical outcomes. Nephrol. Dial. Transplant..

[28] Schwartz GJ (2009). New equations to estimate GFR in children with CKD. J. Am. Soc. Nephrol..

[29] Mi J (2010). Development of blood pressure reference standards for Chinese children and adolescents. Chin. J. Evid. Based Pediatr..

[30] Kidney Disease: Improving Global Outcomes (KDIGO) Acute Kidney Injury Work Group. (2012). KDIGO clinical practice guideline for acute kidney injury. Kidney Int. Suppl..

[31] Subspecialty Group of Renal Diseases, the Society of Pediatrics, Chinese Medical Association. (2017). [Evidence-based guidelines for diagnosis and treatment of primary IgA nephropathy (2016)]. Zhonghua. Er. Ke. Za. Zhi..

[32] Desquilbet L, Mariotti F (2010). Dose-response analyses using restricted cubic spline functions in public health research. Stat. Med..

[33] Croxford, R. *Restricted Cubic Spline Regression: A Brief Introduction*. https://support.sas.com/resources/papers/proceedings16/5621-2016.pdf.

[34] Lausen B, Hothorn T, Bretz F, Schumacher M (2004). Assessment of optimal selected prognostic factors. Biom. J..

[35] Hothorn T, Lausen B (2003). On the exact distribution of maximally selected rank statistics. Comput. Stat. Data.

[36] Worawichawong S (2016). Urine epidermal growth factor, monocyte chemoattractant protein-1 or their ratio as biomarkers for interstitial fibrosis and tubular atrophy in primary glomerulonephritis. Kidney Blood Press Res..

[37] Mattila AL, Viinikka L, Saario I, Perheentupa J (1988). Human epidermal growth factor: renal production and absence from plasma. Regul. Pept..

[38] Kok HM, Falke LL, Goldschmeding R, Nguyen TQ (2014). Targeting CTGF, EGF and PDGF pathways to prevent progression of kidney disease. Nat. Rev. Nephrol..

[39] Sun Y (2021). Epidermal growth factor protects against high glucose-induced podocyte injury possibly via modulation of autophagy and PI3K/AKT/mTOR signaling pathway through DNA methylation. Diabetes Metab. Syndr. Obes..

[40] Lechner J (2007). Opposing roles of EGF in IFN-alpha-induced epithelial barrier destabilization and tissue repair. Am. J. Physiol. Cell Physiol..

[41] Bayram RO, Ozdemir H, Emsen A, Turk Dagi H, Artac H (2019). Reference ranges for serum immunoglobulin (IgG, IgA, and IgM) and IgG subclass levels in healthy children. Turk. J. Med. Sci..

[42] Pottel H (2016). An estimated glomerular filtration rate equation for the full age spectrum. Nephrol. Dial. Transplant..

[43] Norman ME, Gall EP, Taylor A, Laster L, Nilsson UR (1975). Serum complement profiles in infants and children. J. Pediatr..

[44] Liu D (2019). Serum immunoglobulin G provides early risk prediction in immunoglobulin A nephropathy. Int. Immunopharmacol..

[45] Wu D (2021). Mesangial C3 deposition and serum C3 levels predict renal outcome in IgA nephropathy. Clin. Exp. Nephrol..

[46] Ishiguro C (2002). Serum IgA/C3 ratio may predict diagnosis and prognostic grading in patients with IgA nephropathy. Nephron.

[47] Kim SJ (2012). Decreased circulating C3 levels and mesangial C3 deposition predict renal outcome in patients with IgA nephropathy. PLoS One.

[48] Lang YY (2021). Serum IgA/C3 ratio and glomerular C3 staining predict progression of IgA nephropathy in children. Transl. Pediatr..

[49] Tang F, Hu HF, Xu RC, Tao C, Wan QJ (2020). Association between serum IgG concentrations and prognosis in IgA nephropathy. Iran. J. Kidney Dis..

[50] Dong J (2018). A pilot and comparative study between pathological and serological levels of immunoglobulin and complement among three kinds of primary glomerulonephritis. BMC Immunol..

[51] Cortvrindt C, Speeckaert R, Delanghe JR, Speeckaert MM (2022). Urinary epidermal growth factor: a promising “next generation” biomarker in kidney disease. Am. J. Nephrol..

