# Supplementary material for: Urinary epidermal growth factor predicts complete remission of proteinuria in Chinese children with IgA nephropathy
Source: Pediatr Res. 2023 Mar 2;94(2):747–55. doi: 10.1038/s41390-023-02542-0 (PMC10382307; doi:10.1038/s41390-023-02542-0)
Supplement: Supplementary file 1 — Supplementary Materials [file 41390_2023_2542_MOESM1_ESM.pdf]

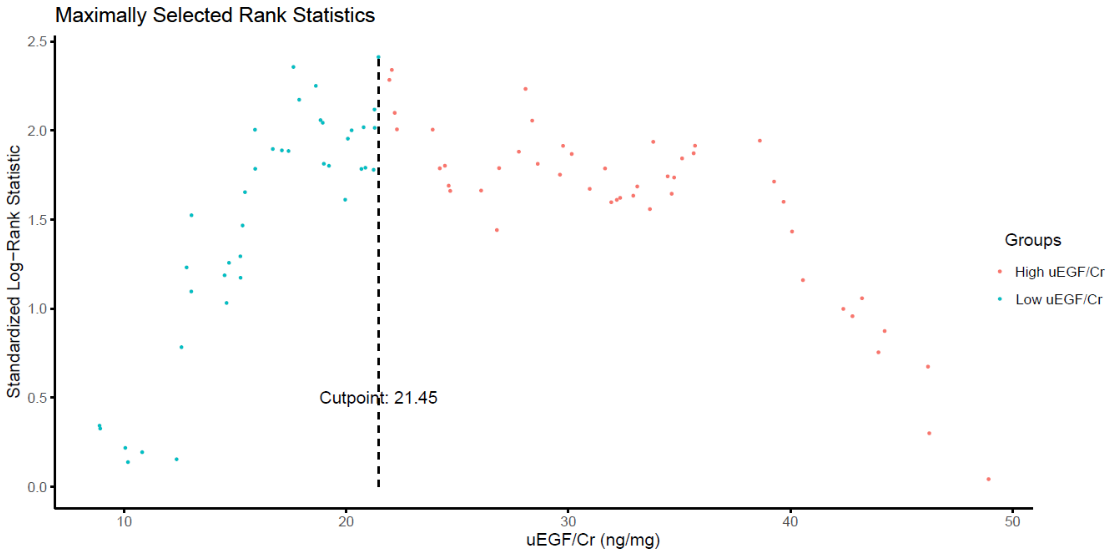

Fig. S1. Maximally selected rank statistics for identifying the optimal cut-off value of the baseline uEGF/Cr. The standardized log-rank statistic was computed for every potential cutoff point. The cutoff point that provided the best separation of CR of proteinuria into two groups, where the standardized statistics reached their maximum, was selected as the cutoff point.

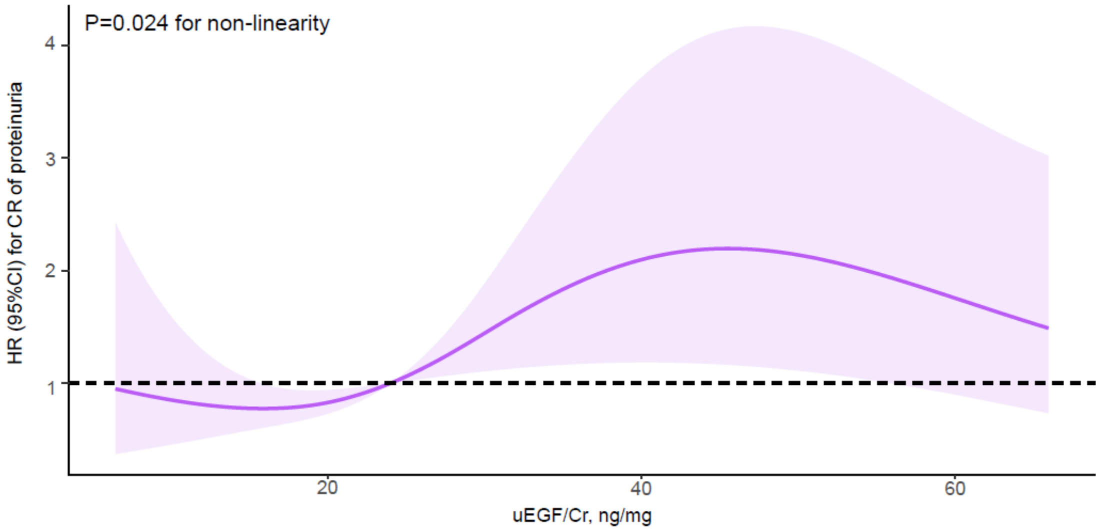

Fig. S2. The non-linear association between uEGF/Cr as a continuous variable and CR of proteinuria according to restricted cubic spline with four knots. The reference value (HR=1) was set at the 50th percentile of uEGF/Cr. The solid line represented HR for the association of uEGF/Cr index with CR of proteinuria, and the shaded portion represented 95% CI.

Table S1. The association of baseline uEGF/Cr with CR of proteinuria after missing values were imputed using multiple imputation with ten imputation datasets (n=108).

	Patients at risk	Univariate		Multivariate [c]	
		HR (95% CI)	P value	HR (95% CI)	P value
Low baseline uEGF/Cr group[a]	49	Reference	-	Reference	-
High baseline uEGF/Cr group[b]	59	1.85 (1.1-3.09)	0.021	2.92 (1.44-5.94)	0.004

a: uEGF/Cr ≤ 21.45 ng/mg. b: uEGF/Cr > 21.45 ng/mg. c: adjusted for age, 24-h UP, serum albumin, gross hematuria, S score, serum IgG levels, and drug usage.

Table S2. The association of baseline uEGF/Cr with CR of proteinuria after patients with AKD were excluded (n=86).

	Patients at risk	Univariate		Multivariate [c]	
		HR (95% CI)	P value	HR (95% CI)	P value
Low baseline uEGF/Cr group[a]	30	Reference	-	Reference	-
High baseline uEGF/Cr group[b]	56	2.55 (1.29-5.03)	0.007	3.68 (1.31-10.28)	0.013

a: uEGF/Cr ≤ 21.45 ng/mg. b: uEGF/Cr > 21.45 ng/mg. c: adjusted for age, 24-h UP, serum albumin, gross hematuria, S score, serum IgG levels, and drug usage.

Table S3. The association of baseline uEGF/Cr with CR of proteinuria after patients with prior therapy were excluded (n=100).

	Patients at risk	Univariate		Multivariate [c]	
		HR (95% CI)	P value	HR (95% CI)	P value
Low baseline uEGF/Cr group[a]	45	Reference	-	Reference	-
High baseline uEGF/Cr group[b]	55	1.91(1.12-3.24)	0.017	1.96 (0.89-4.28)	0.093

a: uEGF/Cr ≤ 21.45 ng/mg. b: uEGF/Cr > 21.45 ng/mg. c: adjusted for age, 24-h UP, serum albumin, gross hematuria, S score, serum IgG levels, and drug usage.
